# Seventy years of data from the world’s longest grazed and irrigated pasture trials

**DOI:** 10.1038/s41597-021-00841-x

**Published:** 2021-02-10

**Authors:** Rich. W. McDowell, R. A. Moss, C. W. Gray, L. C. Smith, G. Sneath

**Affiliations:** 1 AgResearch, Lincoln Science Centre, Private Bag 4749, Christchurch, 8140 New Zealand; 2 Faculty of Agriculture and Life Sciences, Lincoln University, P O Box 84, Lincoln, 7647 Christchurch New Zealand; 3 AgResearch, 204 Woodlands-Morton Mains Road RD 1, Invercargill, New Zealand; 4 Fertiliser Association of New Zealand, Ballinger Building, 58 Victoria Street, Wellington, 6011 New Zealand

**Keywords:** Plant breeding, Biodiversity, Element cycles, Geochemistry, Agroecology

## Abstract

Pastures are the most widespread land use, globally. The Winchmore trials were established in 1948–1949 in Canterbury, New Zealand and examined either different rates of phosphorus (P) fertiliser on the same irrigation schedule (Fertiliser trial), or different irrigation scheduling at the same rate of P application (Irrigation trial). About 96,000 records of soil chemistry and physical data and pasture yield and botanical composition are available along with nearly 7000 soil samples. These data have been used in 475 publications that have explored topics as diverse as: improvements in sheep, dairy and deer production; the efficacy and scheduling of irrigation; improvements in pasture and crop production; agronomic and environmental soil and water research; and entomology. In addition to above topics, these data are invaluable for calibrating models to predict long-term issues like the accumulation of soil carbon or contaminants like cadmium and informing policy on climate change and agricultural practices. The data and soil samples are available for use and may yet yield discoveries, unforeseen 70 years ago.

## Abstract (summary)

**Table:** 

Measurement(s)	phosphorus • cadmium • soil moisture • composition of soil • acidity of soil • total biomass yield • porosity of soil • hydraulic conductivity • temperature of soil
Technology Type(s)	colorimetry • inductively coupled plasma-atomic emission spectrometry • gravimetric analysis • pH meter • Calculation • ring infiltrometer • Thermometer Device
Factor Type(s)	fertiliser application rate • irrigation scheduling • year of data collection
Sample Characteristic - Environment	farm soil • pasture • irrigation ditch • leachate
Sample Characteristic - Location	New Zealand

Machine-accessible metadata file describing the reported data: 10.6084/m9.figshare.12996692

## Background & Summary

The Winchmore field trials are the longest running trials of grazed and irrigated pasture, globally^1^. The initial aims of the experiments were to: (1) establish the response of pasture (ryegrass and white clover) production (kg ha^−1^ yr^−1^) and productivity (production per unit of phosphorus input) to increasing rates of phosphorus (P) fertiliser applied as single superphosphate (SSP) or reactive phosphate rock (RPR), and (2) measure the response of pasture to different rates of irrigation at the same rate of SSP. Since their establishment in 1949 for the irrigation trial and 1952 for the fertiliser trial, the data have laid the foundation of productive irrigation systems that earn several billions in revenue annually in New Zealand and overseas and provisioned data to support 475 publications^2^.

The initial focus on production and productivity revealed important findings on the optimal management of productive pastures. For instance, in response to P applications, the rate of organic P accumulation increased quickly and then reached a plateau, presumably as all plant-P is supplied from inorganic sources. This isolated an agronomic optimum Olsen P concentration for pasture production at 15–20 mg kg^−1^, mostly as inorganic P^3^. Additional data showed that this concentration allowed the proportion of ‘P-hungry’ nitrogen (N)-fixing legumes in the sward that was economically optimal and supported increased soil biodiversity^4–7^. However, ancillary trial data also showed that rapid declines in pasture production were possible if P applications were stopped, for example, in response to short-term price rises, such as the 800% increase in P fertiliser prices seen in 2008^8,9^.

More recently the focus has shifted to environmental issues such as water and soil quality. The trial’s range of soil Olsen P concentrations was used to show an increase in P losses in runoff (up to 7 kg P ha^1^ yr^−1^) in response to flood irrigation^10^. The loss of P increased exponentially beyond an Olsen P of 25 mg kg^−1^ affirming advice that exceeding the agronomic optimum represents an unnecessary risk to surface water quality^3^. The analysis of soils in the Winchmore plots over time has quantified how fast soils are accumulating cadmium (Cd) applied as a by-product of SSP and RPR application^11,12^. Subsequent modelling has led to national policies to decrease Cd-concentrations in P-fertilisers and minimise the risk of Cd contaminating livestock^13–15^. Additional work included these topsoils along with a range of other soils from around New Zealand of high fertility but different rainfall and hypothesized that while initially accumulating carbon (C), concentrations had reached a plateau and the higher Olsen P soils were potentially losing C^16^. This would have impacted upon the accuracy of national inventories for CO_2_ emissions; however, subsequent research established that over the depth of the profile (1-m), C stocks were not different between the different fertiliser treatments but was if compared to an unirrigated treatment where poorer pasture quality and cycling allowed C to accumulate^17–19^.

The original station operators had the vision to archive samples of soil, pasture and fertiliser, recognising that advances in analytical techniques may lead to new discoveries. The number of archived soil samples now approaches 7000. Samples of the SSP and RPR fertiliser applied have also been kept since 1998. However, the original archived pasture samples have been lost due to insect damage during storage. Records were also kept of sowing rates and harvest dates, climatic conditions, soil moisture, and monthly pasture production and botanical composition. As part of this publication, these paper records have now been put into electronic format. In this paper we outline the context, structure and potential uses of the data from the Winchmore trials with a view that they be used by the global scientific community.

## Methods

### Experimental design

**Fig. 1:**
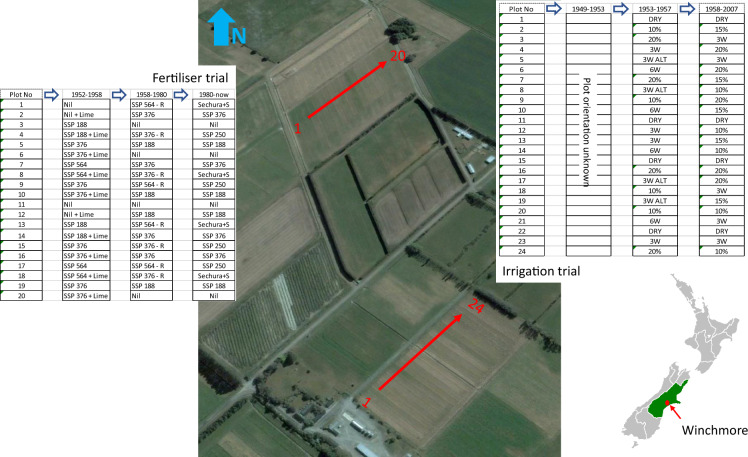
Location of Winchmore within the Canterbury region (coloured green) and the layout of the long-term fertiliser and irrigation trials over time.

The Winchmore Irrigation Research Station is in the centre of the Canterbury plains, the largest area of flat land in New Zealand (43.787° S, 171.795° E; Fig. 1). It is at an altitude of 160 m above sea level, a mean annual temperature of 12 °C, and has an annual rainfall of 745 mm (range 491–949 mm)^20^. The soil is a Lismore stony silt loam classified as an Orthic Brown soil in the New Zealand soil classification and as an Udic Ustochrept in USDA soil classification^21^. Flood irrigation, known locally as border-check/dyke irrigation, was installed at the site in 1947. However, the two long-term trials, hereafter known as the fertiliser and irrigation trials, were established in 1952 and 1949, respectively.

Full details of the setup of the fertiliser and irrigation trials between 1949–1951, including the political rationale for the trial, its statistical design, cultivation dates, sowing rates of perennial ryegrass (*Lolium spp)* and white clover (*Trifolium repens)* and initial fertiliser and irrigation treatments are available elsewhere^20^.

The fertiliser trial has 20 border check irrigation bays divided into five treatments each with four replicates set out in a randomised block design (Fig. 1). From 1952/53 to 1957/1958 treatments were: nil (no P applied), 188, 376 (annually and split P applications), and 564 kg SPP ha^−1^. All P applications occurred annually in autumn except for the 376 kg SSP ha^−1^ treatment which had two treatments divided into an annual autumn application and split applications in between autumn and spring. From 1958/59 to 1979–80 the nil and 188 and 376 (split autumn and spring application) SSP treatments were unaltered, while P applications were stopped to the annual 376 and 564 SSP treatments. In 1972, 4.4 t/ha of lime (caclium carbonate) was applied to all treatments^22^. From 1980 onwards the nil, and 188 SSP treatments and the 376 SSP treatment, now receiving winter fertiliser applications, were joined by a treatment applying 250 SSP ha^−1^ in winter to the previous 376 SSP treatment and a Sechura rock phosphate treatment applying 22 kg P ha^−1^ in winter to the former 576 SSP treatment.

Each irrigation bay was fenced off, 0.09 ha in size and grazed by separate mobs of sheep at 6, 11, and 17 stock units (with one stock unit equivalent to one ewe at 54 kg live-weight) per replicate for the nil, 188 SSP, and 376 SSP treatments, respectively. This separation prevented carry-over of dung P and other nutrients and contaminants between treatments. No grazing occurred in winter. Flood irrigation was applied when soil moisture content (w w^−1^) fell below 15% (0–100 mm depth). This occurred on-average 4.3 times per year.

The irrigation trial had 24 irrigation bays (each 0.09 ha in size) which had lime applied to the whole trial in 1948 (5 t ha^−1^) and 1965 (1.9 t ha^−1^) to maintain soil pH at 5.5–6.0. From 1951 to 1954 treatments were SSP applied at 250 kg ha^−1^ in autumn annually and either four replicates of dryland, or five replicates of irrigation applied at one, two, three, six-weekly intervals or at three-weekly intervals in alternate seasons. From 1953/54 to 1956/57 the weekly and two-weekly treatments were replaced by irrigation when soil moisture in the top 100-mm of soil reach 50 and 0% available soil moisture (asm), respectively. In 1958 the irrigation trial was cultivated with a rotary hoe and grubber, 140 kg SSP ha^−1^ applied and the site re-sown in ryegrass and white clover. From 1958/59–2007 the site had the same blanket application of SSP and four replicates of dryland, while a completely randomised design was used to impose five replicates of four treatments (Fig. 1) that looked at irrigation applied when soil moisture in the top 100-mm of soil reach 10, 15 and 20% (equivalent to 50% asm with 0% asm being wilting point) and irrigation on a 21-day interval. The need for irrigation to the irrigation and fertiliser trials was informed by soil moisture measured weekly by technical staff using a mixture of gravimetric analyses (1950–1985), neutron probe (1985–1990) and time-domain reflectometer (1990-onwards). Irrigation was applied at a rate of 100 mm per event^20^.

Except for winter, when no grazing occurred, each treatment was rotationally grazed by a separate flock of sheep with 6 and 18 stock units per replicate for the dryland and 20% v/v treatments, respectively.

The irrigation trial finished in October 2007 although the P fertiliser regime continued. All irrigated treatments shifted to the same three weekly schedule as the long-term Fertiliser trial. The dryland treatment remained unirrigated. The Winchmore farm was converted into a commercial irrigated farm operation and sold in 2018. The fertiliser trial was also sold but with a covenant ensuring it continues to operate as per normal except that irrigation from 2018 onwards is now applied by spray irrigation with the aim of ensuring soil moisture is maintained above 90% of field capacity. Since January 2019 there are daily soil moisture meter records from a moisture meter installed into one of the control plots. Soil moisture, rainfall and irrigation are recorded.

**Fig. 2:**
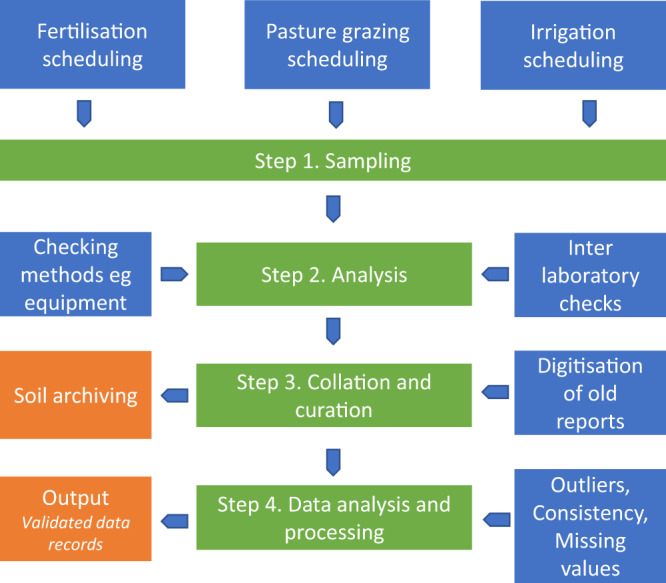
Flowchart of the steps involved in sampling, analysis, collation and curation and data analysis and processing of the databases from the Winchmore Trials. Note that blue and orange boxes are sub tasks associated with each step and resulting outputs, respectively.

The production of the Winchmore trials data records^23^ involved a three-step process (Fig. 2).

### Step 1: Soil and pasture sampling

Pasture production was measured from two exclusion cages (3.25 m long × 0.6 m wide) per plot^24^. Areas within each cage were trimmed to 25 mm above ground level and left for a standard grazing interval for that time of year. Following grazing a lawnmower was used to harvest a 0.40 m wide strip in the middle of each enclosure to 25 mm above ground level. The wet weight was determined, and a sub-sample taken to determine dry matter content with a separate sample manually dissected into grass, clover and weeds. All surplus mown herbage was returned to the plot. Approximately 9–10 cuts were made annually. A composite soil sample of 10 cores (2.5 cm diameter and 7.5 cm deep) was collected from each plot. These were collected four times annually (July, prior to fertiliser application, and October, January and April), using established best practices^24,25^. In 2009 soil samples were also collected from the 0–75, 75–150, 150–250, 250–500, 500–750, and 750–1000 mm depths on both trials^17^. During 2018, prior to cultivation, soil on the unirrigated, 10 and 20% soil moisture treatments of the irrigation trial were sampled at 0–150, 150–250, 250–500, 500–750, 750–1000, 1000–1500, and 1500–2000 mm depths. The top 250 mm of these samplings were collected by hand using an auger, but deeper depths were excavated via a mechanical digger. Representative sub-samples were taken from each depth. Annual samplings were crushed, dried and sieved <2 mm for storage and later chemical analysis. The depth samplings were crushed, dried and sieved <6 mm for storage and chemical analysis. The mass of stones was recorded during sieving for the depth sampling.

In February 2002 and August 2007 10 cm diameter and 5 cm deep rings were taken in duplicate of the Irrigation trial for later soil physical analyses.

During 2017 soil on all treatments of the fertiliser trial was sampled using a soil corer to 0–75, 75–150, and 150–300 mm depths at five equally spaced distances centrally located down the irrigation bay. A further series of soil samples were obtained from the fertiliser trial during 2018 from the nil, 188 and 376 kg SSP ha^−1^ yr^−1^ treatments at 0–75 and 75–175 mm depths at five equally spaced distances down and five locations across the irrigation bay.

### Step 2: Soil and fertiliser analyses

Soil chemical samples were commonly analysed for Truog P (1952–1981)^26^, Olsen P concentration (1976-onwards)^27^, pH in water^28^, exchangeable cations (potassium (K), magnesium (Mg), calcium (Ca)^29^, sulphate-sulphur (S), and occasionally organic S^30^, reserve K^31^, inorganic P^32^, organic C^33^, organic matter^33^ and total C^34^, Cd^35^, P^36^, fluorine (F)^37^, N^38^ and uranium (U)^11^. Stored samples of the fertilisers applied from 1998–2010 were also digested and analysed via inductively coupled plasma-optical emission spectroscopy (ICP-OES)^11^. Several quality assurance checks were made of these analysis and soil moisture measurements (see Technical Validation section). Soil physical measurements of porosity, bulk density, particle size (proportions of sand, silt and clay), and hydraulic conductivity^39^ were made for samples taken in 2002 and 2007 from the Irrigation trial. Some assessments were made of soil biological diversity in the Irrigation trial (microbial, fungal and invertebrate communities)^6,7^.

### Step 3: Collation and curation

Data were sourced from several technical reports and published articles (Table 1). All data in reports written prior to 1995 had to be digitised before being entered into Excel spreadsheets. Data were converted via a commercial data capture service who used an XML-based data conversion tool, ImageXP with an estimated accuracy of 99.995%^40^. In addition to records, archived soil samples, each with a unique identifier outlining the trial, year and season of sampling, sample depth and replicate plot number have been stored in a soil archive based at AgResearch’s Ruakura campus in Hamilton, New Zealand. Soil samples exist for approximately 85% of years sampled for the fertiliser and (up to 2007) irrigation trials. Samples of the fertilisers applied to both trials exist in the archive for 1997, 1998, 1999, 2000, 2001, 2008, 2009, 2010, 2011, 2012, 2013, 2014, 2015, 2016, 2017, 2018 and 2019.

**Table 1:** Summary of the data files available at Figshare^23^.

File ID	Trial	Description of content (number of samples)
Fertiliser trial soil chemistry data.xlsx	Long-term fertiliser trial 1952–2019	• Data from 1952–2019 for soil pH, exchangeable cations (Ca, K, Na, Mg) and available P (n = 25404). • Occasional data from 1952–2015 for organic S, reserve K, inorganic P, and total Cd, F, U, S, N and C (n = 1557). • Analysis of total concentrations of other elements in fertilisers applied between 1998 and 2010 (n = 356).
Irrigation trial soil chemistry data.xlsx	Long-term irrigation trial 1951–2018	• Data from 1956–2018 for soil pH, exchangeable cations (Ca, K, Na, Mg) and available P (n = 16430). • Occasional data from 1951–2018 for organic S, reserve K, inorganic P, and total S, N, C, P, Cd and U and organic C and organic matter (n = 989).
Fertiliser trial pasture production and composition data.xlsx	Long-term fertiliser trial 1952–2019	• Monthly mean dry matter yield for each treatment 1980–2019 (n = 3116). • Annual dry matter yield for each plot from 1994–2019 (n = 494). • Seasonal or annual botanical composition and yield from 1952–2019 (n = 3351) and from each plot and cut from 2005–2019 (n = 2944).
Irrigation trial pasture production and composition data.xlsx	Long-term irrigation trial 1960–1985	• Monthly and annual mean dry matter yield for each treatment 1960–1985 (n = 9010). • Annual botanical composition from 1962–1985 (n = 4080) and for the main species (n = 120).
Irrigation trial soil physics, irrigation schedule, soil moistures and temperatures.xlsx	Long-term irrigation trial 1951–2018	• Measurements of bulk density, porosity, saturated and unsaturated hydraulic conductivity for all plots in 2002 and 2007 (n = 1169). • Particle size measured in 2007 for all plots (n = 94). • Irrigation scheduling from 1952–2002 for all treatments (n = 840). • Monthly mean soil moisture measurements 1952–1986 for all treatments (n = 2352). • Weekly mean soil moisture measurements from 1980–2003 for all treatments (n = 7006). • Monthly maximum and mean soil temperatures for each treatment (n = 1734). • Daily rainfall recorded on site from 1966–2007 (n = 15187).

### Step 4: Data analysis and processing

#### Removal of outliers

We used methods outlined in the literature to check our data for outliers^41^. We established an acceptable range for daily temperature of −20 to +40 and a daily maximum rainfall of 100 mm as these were consistent with long-term meteorological data collected since 1950. Acceptable ranges for soil moisture, soil Olsen P, K, Mg, Ca and S concentrations were set at 0–100% asm, 1–200 mg Olsen P kg^−1^, 0.5–12 me Ca 100 g^−1^, 0.1–5 me K 100 g^−1^, 0.1–4 me Mg 100 g^−1^, and 1–120 mg S kg^−1^. The soil chemical concentrations, for example for Olsen P, corresponded to the maximum that were unlikely to indicate contamination by dung or fertiliser^42^. An upper range for pasture harvest data was set at 5,000 kg ha^−1^. Data outside these temperature, moisture, soil Olsen P concentration and pasture harvest ranges were discarded. For soil total Cd, concentrations below a detection limit of 0.5 µg kg^−1^ were recorded as 0.25 µg kg^−1^. Checks are continuing for regular soil analysis of K, Mg, Ca or S, while no checks are planned for other soil data owing to their infrequent measurement.

We calculated the mean standard deviation (SD) for soil Olsen P, S, and exchangeable Ca, K, and Mg concentrations in decadal intervals from 1960 and flagged those values that were >± 3SDs away from the mean. These data were inspected manually and excluded if variation was not consistent with other replicate plots. This removed <0.3% of the data.

#### Consistency

We compared weekly soil moisture across replicates and treatments to determine if irrigation events had occurred as the treatment design called.

#### Missing values

Our dataset contains some missing values that cause gaps in our data records. These were caused by either no sampling or a loss of data records. Missing values are indicated by blanks in our dataset. In the Fertiliser trial they constituted ~18% of pH and exchangeable cations data for 36 sampling dates and 9% of Olsen P data for 20 sampling dates. However, means were available for most of these dates. In the Irrigation trial, 4% of pH and exchangeable cations data for 6 sampling dates and 5% of Olsen P data for 7 sampling dates. Unlike the Fertiliser trial, means were unavailable for these missing dates.

#### Future content

The intent of the Winchmore database is for it to be kept live. Updates will be made to the Figshare repository^23^ as and when new data become available. Additional reports on annual soil chemical analyses and pasture production and botanical composition for the Fertiliser trial are available at www.fertiliser.org.nz.

## Data Records

Data are published in five spreadsheets at Figshare^23^ beginning wither either “Fertiliser trial…” or “Irrigation trial…”. There are two files containing the soil chemistry data for the Fertiliser and Irrigation trials and two files containing pasture production and botanical composition data. Data are also available for irrigation schedules and soil moisture, temperature and physical measures for the Irrigation trial. Note that the Fertiliser trial was irrigated on the same three-weekly schedule as the Irrigation trial. All files have information concerning the trial design and metadata included in the first two tabs.

**Fig. 3:**
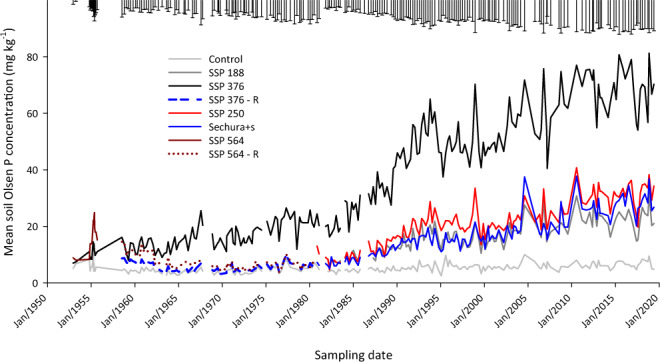
Mean soil Olsen P concentrations for the long-term fertiliser trial from 1951–2019 for each treatment. Error bars are the 95% confidence interval for the comparison of treatment means.

A summary of the nearly 70-year record of data is given in Table 1 and example of the Olsen P data for the Fertiliser trial in Fig. 3., showing the gradual increase in soil Olsen P concentrations over time.

## Technical Validation

### Storage

The long-term storage of air-dried soils is known to affect some chemical parameters such as a decrease in pH caused by the hydrolysis of Al^3+^, especially in acid soils when stored in periodically humid air^43^ and an increase in exchangeable Na (if soils are stored in Na-based glassware^44^. Other parameters like total C show no change while no data is available for Olsen P, sulphate-S, organic S, or total Cd and U. Fortunately, the Winchmore samples have been stored in paper bags in a naturally low humidity environment (at Winchmore) until 2004 and in 200 mL sealed plastic containers at ambient temperature at AgResearch-Ruakura (Hamilton, New Zealand) since then. Nevertheless, we cannot guarantee that some chemical parameters have not changed during storage.

### Validating variation between methods and laboratories

Several of the chemical parameters were validated against reference soils and their methods checked for compatibility over time and between laboratory variation. The use of Truog P until 1976 required data to be converted into Olsen P. An overlap from 1976–1981 allowed a regression equation to be generated to predict Olsen P with a high degree of confidence (Olsen P = 1.376Truog P + 0.06; R^2^ = 0.91, *P* < 0.001, n = 118, mean Olsen P = 8.5 and range = 1–28 mg kg^−1^). Before changing laboratories in the late 1980’s and early 2000’s to test the regularly analysed parameters Olsen P and exchangeable cations, the same subsample of 30 archived soils selected at random from 1960–1980 were sent to the two laboratories and the data checked to see if the variation between mean concentrations was <5%. The same 5% threshold was used to check soil moisture measurements using neutron probes or TDR against the more laborious, but ‘true’ measurements of soil moisture via the gravimetric method.

Measurements of pasture production at the site had been conducted largely by one person for the last 50 years. This person was trained by the original operators of the sites during a brief hand-over period. The same pasture cages have been used since the inception of both trials. A drum-type mower has been used to cut herbage from the trials’ beginning until 1998 from which time a rotary mower has been used.

Of the irregularly measured chemical parameters, total Cd was determined by different machines. Initial measurement using ICP-OES^11^ yielded more erratic data, especially at higher concentrations, than more recent assessments of the same soils using inductively coupled plasma-mass spectroscopy (ICP-MS)^35,45^. We include only the latter measurements of total Cd in our dataset as these were included in measurement runs that included checks for Cd concentration in reference soils were no more than 5% greater than the known total Cd concentration^35^.

## Usage Notes

### Use

Data from the Winchmore trials have been used in a wide range of academic areas and subsequent extension efforts with farmers. A comprehensive bibliography published in 2012^2^ lists such areas to include journal articles and conference presentations. We grouped these into five broad classes:

1. Improvements in sheep, dairy and deer production (n = 49), which included the establishment and refinement of stocking rates, carrying capacities, feed requirements, nutrient deficiencies, improvements in sheep fertility and liveweight gain and mixed goat and sheep grazing^46–48^.
2. The efficacy and scheduling of irrigation (n = 50), which included engineering requirements for and maintenance of flood irrigation systems, and water use efficiency and the effect on groundwater recharge – leading to the gradual phasing out of flood irrigation to spray irrigation in New Zealand for environmental and financial reasons^49,50^.
3. Improvements in pasture and crop production (n = 184) which drew from the long-term trials to influence the design and trialling of cereal and legume production trials, the role of long-term no till and crops for ethanol production^51,52^.
4. Agronomic and environmental soil research (n = 94) that focused on soil moisture levels in response to irrigation over time and modelling fertiliser requirements and losses in response to pasture performance in addition to the aforementioned research into contaminant metals like Cd or environmental issues such as nutrient losses via runoff and leaching^39,53,54^.
5. Entomology (n = 14) including the long-term monitoring of the lifecycle and impact of grass grub beetle (*Costelytra zaelandica*) on pasture growth, the presence and behaviour of earthworms in irrigated and dryland pastures, and the presence of insecticide residues such as DDT in the soil^7,55,56^.

Additional technical reports and miscellaneous publications numbered 43. A search of the Ovid database indicated an additional 44 journal publications since 2012, totalling 475 publications over 70 years of which 94 were in the international literature. This is a similar number over the last 8 years to that produced from the more well-known, and longest running grassland trial in the world, Park Grass at the Rothamsted Research Station in the UK (n = 55; 2007–2016)^57^. This similarity emphasizes the value of Winchmore as a long-term site of national and potentially global interest.

The future of the long-term fertiliser trial has been secured with a lease in place to the Fertiliser Association of New Zealand out to 2052. The site is managed by AgResearch on behalf of the Association. The long-term lease and a commitment to provide the data under the CC-BY licence will see it used wider and for questions that only long-term trials and the archiving of samples can answer. Such questions include, but are not limited to:

- defining the concentration of Cd in P fertiliser that can stop or reduce an increase soil total Cd content;
- the impact on nutrient cycling associated with increasing CO_2_ concentrations over decadal timeframes; and
- isolating the biodiversity, function and connections between the soil-plant-animal microbiome.

### Data and sample access

The data are available but remain the property of the funders. Users of the data are encouraged to cite its origin (this paper) and the three funding bodies (the New Zealand Government, AgResearch Ltd, and the Fertiliser Association of New Zealand). All of the data files are supplied in read-only form to avoid users unintentionally compromising files. We also encourage users to fill out the Data Use Form. We will use the information provided to track use of the database. Your details will be kept private.

Soil and fertiliser samples may also be available on request by emailing the database managers at winchmore@agresearch.co.nz. We will only consider sending samples to users who have lodged a Data Use Form with us. Given their finite volume, we may require further information to judge the value of the work before samples would be released. Samples would only be provided on the understanding that any analysis data so gained will be available to be archived in this database.

## Data Availability

No code was used to generate the data records.

## References

[1] McDowell R, Smith C (2012). The Winchmore Trials. N. Z. J. Agric. Res..

[2] Cousins KA, McDowell RW (2012). Bibliography of research from the Winchmore Irrigation Research Station, Canterbury, New Zealand: 1951 to 2011. N. Z. J. Agric. Res..

[3] McDowell RW, Condron LM (2012). Phosphorus and the Winchmore trials: review and lessons learnt. N. Z. J. Agric. Res..

[4] Smith LC, Moss RA, Morton JD, Metherell AK, Fraser TJ (2012). Pasture production from a long-term fertiliser trial under irrigation. N. Z. J. Agric. Res..

[5] Macintosh KA (2019). Transforming soil phosphorus fertility management strategies to support the delivery of multiple ecosystem services from agricultural systems. Sci. Total Environ..

[6] Wakelin SA (2016). Analysis of soil eDNA functional genes: potential to increase profitability and sustainability of pastoral agriculture. N. Z. J. Agric. Res..

[7] Fraser PM, Schon NL, Piercy JE, Mackay AD, Minor MA (2012). Influence of summer irrigation on soil invertebrate populations in a long-term sheep irrigation trial at Winchmore (Canterbury). N. Z. J. Agric. Res..

[8] McBride SD, Nguyen ML, Rickard DS (1990). Implications of ceasing annual superphosphate topdressing applications on pasture production. Proc. N. Z. Grass. Assoc..

[9] Khabarov, N. & Obersteiner, M. Global Phosphorus Fertilizer Market and National Policies: A Case Study Revisiting the 2008 Price Peak. *Frontiers in Nutrition* **4** (2017). 10.3389/fnut.2017.00022 PMC5469897 28660192

[10] McDowell RW, Rowley D (2008). The fate of phosphorus under contrasting border-check irrigation regimes. Soil Res..

[11] McDowell RW (2012). The rate of accumulation of cadmium and uranium in a long-term grazed pasture: implications for soil quality. N. Z. J. Agric. Res..

[12] Gray CW, McLaren RG, Roberts AHC, Condron LM (1999). The effect of long-term phosphatic fertiliser applications on the amounts and forms of cadmium in soils under pasture in New Zealand. Nutrient Cycling in Agroecosystems.

[13] Cadmium Working Group. Cadmium and New Zealand Agriculture and Horticulture: A Strategy for Long Term Risk Management. 27 (Ministry of Agriculture and Forestry, Wellington, New Zealand, 2011).

[14] Abraham E (2020). Cadmium in New Zealand agricultural soils. N. Z. J. Agric. Res..

[15] Salmanzadeh M (2017). Isotope Tracing of Long-Term Cadmium Fluxes in an Agricultural Soil. Environ. Sci. Technol..

[16] Schipper LA (2007). Large losses of soil C and N from soil profiles under pasture in New Zealand during the past 20 years. Global Change Biol..

[17] Condron LM, Black A, Wakelin SA (2012). Effects of long-term fertiliser inputs on the quantities of organic carbon in a soil profile under irrigated grazed pasture. N. Z. J. Agric. Res..

[18] Kelliher FM, Condron LM, Cook FJ, Black A (2012). Sixty years of seasonal irrigation affects carbon storage in soils beneath pasture grazed by sheep. *Agric*. Ecosyst. Environ..

[19] Schipper LA (2013). Decadal Changes in Soil Carbon and Nitrogen under a Range of Irrigation and Phosphorus Fertilizer Treatments. Soil Sci. Soc. Am. J..

[20] Rickard DS, Moss RA (2012). Winchmore and the long-term trials: the early history. N. Z. J. Agric. Res..

[21] Hewitt, A. E. *New Zealand soil classification*. 3rd edn, (Manaaki Whenua Press, Landcare Research, 2010).

[22] Rickard, D. S. & McBride, S. D. Long term application and residual effects of superphosphate and effects of reactive phosphate rock on irrigated pasture. (MAF Winchmore Irrigaton Research Station, Winchmore, Canterbury, New Zealand, 1987).

[23] McDowell RW (2021). figshare.

[24] Lynch, P. B. Conduct of field experiments. 155 (New Zealand Department of Agriculture, Wellington, New Zealand, 1966).

[25] Morton, J. D. & Roberts, A. H. C. *Fertiliser use on New Zealand sheep and beef farms*. 5th edn, (New Zealand Fertiliser Manufacturers’ Research Association, 2016).

[26] Truog E (1930). The determination of readily available phosphorus of soils. Journal of the American Society of Agronomy.

[27] Olsen, S. R., Cole, C. V., Watanbe, F. S. & Dean, L. A. In *United States Department of Agriculture. Circular No. 939* 19 (U.S. Dept. of Agriculture, Washington, D.C., 1954).

[28] Hendershot, W. H., Lalande, H. & Duquette, M. In *Soil sampling and methods of analysis* (ed M. R. Carter) 141–146 (Lewis Publishers, 1993).

[29] Hendershot, W. H., Lalande, H. & Duquette, M. In *Soil sampling and methods of analysis* (ed M.R. Carter) 167–176 (Lewis Publishers, 1993).

[30] Kowalenko, C. G. In *Soil sampling and methods of analysis* (ed M.R. Carter) 231–238 (Lewis Publishers, 1993).

[31] Carey PL, Metherell AK (2003). Rates of release of non-exchangeable potassium in New Zealand soils measured by a modified sodium tetraphenyl-boron method. N. Z. J. Agric. Res..

[32] Tiessen, H. & Moir, J. O. In *Soil sampling and methods of analysis* (ed M.R. Carter) 75–86 (Lewis Publishers, 1993).

[33] Grewal KS, Buchan GD, Sherlock RR (1991). A comparison of three methods of organic carbon determination in some New Zealand soils. J. Soil Sci..

[34] Tiessen, H. & Moir, J. O. In *Soil sampling and methods of analysis* (ed M.R. Carter) 187–200 (Lewis Publishers, 1993).

[35] Gray, C. W., McDowell, R. W. & Noble, A. D. L. Total soil cadmium concentrations in the Winchmore long-term phosphorus fertiliser trial are still increasing. *N. Z. J. Agric. Res*. 1–8 (2020).

[36] Crosland AR, Zhao FJ, McGrath SP, Lane PW (1995). Comparison of Aqua regia digestion with sodium carbonate fusion for the determination of total phosphorus in soils by inductively coupled plasma atomic emission spectroscopy (ICP). Commun. Soil Sci. Plant Anal..

[37] Gray CW (2018). Fluorine in soils under pasture following long-term application of phosphate fertiliser in New Zealand. Geoderma Regional.

[38] McGill, W. B. & Figueiredo, C. T. In *Soil sampling and methods of analysis* (ed M. R. Carter) 201–212 (Lewis Publishers, 1993).

[39] Srinivasan MS, McDowell RW (2009). Irrigation and soil physical quality: An investigation at a long-term irrigation site. N. Z. J. Agric. Res..

[40] DataNZ. *Data Entry Services*, https://www.datanz.com/digitisation-services/transcription-services (2020).

[41] Campbell JL (2013). Quantity is Nothing without Quality: Automated QA/QC for Streaming Environmental Sensor Data. Bioscience.

[42] Roberts, A. H. C. & Morton, J. D. Fertiliser use on New Zealand dairy farms. 52 (New Zealand Fertiliser Manufacturers’ Research Association, Auckland, New Zealand, 2009).

[43] Falkengren-Grerup, U. Effects of Long-Term Storage on Some Chemical Properties of Forest Soil Samples. *Ecological Bulletins*, 129–132 (1995).

[44] Blake L, Goulding KWT, Mott CJB, Poulton PR (2000). Temporal changes in chemical properties of air-dried stored soils and their interpretation for long-term experiments. Eur. J. Soil Sci..

[45] Kelliher FM, Gray CW, Noble ADL (2017). Superphosphate fertiliser application and cadmium accumulation in a pastoral soil. N. Z. J. Agric. Res..

[46] Bray AR, Burton RN, Cox BT (1987). Magnesium supplementation of lactating ewes and lamb growth. N. Z. Vet. J..

[47] Mackay AD (2001). Moving towards low-chemical and caring farming systems. Proc. N. Z. Grass. Assoc..

[48] Moss RA (1976). Sheep production high on irrigated pasture. New Zealand Journal of Agriculture.

[49] Weaver L (2016). Microbial transport into groundwater from irrigation: Comparison of two irrigation practices in New Zealand. Sci. Total Environ..

[50] Jenkins, B. In *Water Pricing Experiences and Innovations* (eds Ariel Dinar, Víctor Pochat, & José Albiac-Murillo) 263–288 (Springer International Publishing, 2015).

[51] Francis GS, Haynes R, Knight TL (1992). An overview of results from the long-term no-tillage trials at Winchmore. Proceedings of the Agronomy Society of New Zealand.

[52] Martin RJ, Drewitt EG, Sinton SM, Tabley FJ, Nicholl AP (1982). Effect of sowing date on the yield and sugar content of sugar beet and fodder beet at four sites. Proceedings of the Agronomy Society of New Zealand.

[53] Edmeades DC, Metherell AK, Waller JE, Roberts AHC, Morton JD (2006). Defining the relationships between pasture production and soil P and the development of a dynamic P model for New Zealand pastures: A review of recent developments. N. Z. J. Agric. Res..

[54] Metherell, A. K., Condron, L. M. & Eden, R. M. In *Proceedings XVIII International Grasslands Congress, Winnipeg, Manitoba* 10 33–10 34 (1997).

[55] Boul HL, Garnham ML, Hucker D, Baird D, Aislabie J (1994). Influence of Agricultural Practices on the Levels of DDT and Its Residues in Soil. Environ. Sci. Technol..

[56] Villalobos FJ, Goh KM, Saville DJ, Chapman RB (1997). Interactions among soil organic matter, levels of the indigenous entomopathogenic bacterium Serratia entomophila in soil, amber disease and the feeding activity of the scarab larva of Costelytra zealandica: a microcosm approach. Applied Soil Ecology.

[57] Perryman SAM (2018). The electronic Rothamsted Archive (e-RA), an online resource for data from the Rothamsted long-term experiments. Scientific Data.

